# Chemoenzymatic access to enantiopure N-containing furfuryl alcohol from chitin-derived N-acetyl-D-glucosamine

**DOI:** 10.1186/s40643-021-00435-w

**Published:** 2021-08-27

**Authors:** Ya-Cheng Hao, Min-Hua Zong, Zhi-Lin Wang, Ning Li

**Affiliations:** 1 School of Food Science and Engineering, South China University of Technology, 381 Wushan Road, Guangzhou, 510640 China; 2 Agro-Biological Gene Research Center, Guangdong Academy of Agricultural Sciences, 20 Jinying Road, Guangzhou, 510640 China

**Keywords:** Asymmetric synthesis, Biobased chemicals, Carbonyl reductases, Enzyme catalysis, Organonitrogen chemicals

## Abstract

**Background:**

Chiral furfuryl alcohols are important precursors for the synthesis of valuable functionalized pyranones such as the rare sugar L-rednose. However, the synthesis of enantiopure chiral biobased furfuryl alcohols remains scarce. In this work, we present a chemoenzymatic route toward enantiopure nitrogen-containing (*R*)- and (*S*)-3-acetamido-5-(1-hydroxylethyl)furan (3A5HEF) from chitin-derived N-acetyl-D-glucosamine (NAG).

**Findings:**

3-Acetamido-5-acetylfuran (3A5AF) was obtained from NAG via ionic liquid/boric acid-catalyzed dehydration, in an isolated yield of approximately 31%. Carbonyl reductases from *Streptomyces coelicolor* (ScCR) and *Bacillus* sp. ECU0013 (YueD) were found to be good catalysts for asymmetric reduction of 3A5AF. Enantiocomplementary synthesis of (*R*)- and (*S*)-3A5HEF was implemented with the yields of up to  >  99% and the enantiomeric excess (ee) values of  >  99%. Besides, biocatalytic synthesis of (*R*)-3A5HEF was demonstrated on a preparative scale, with an isolated yield of 65%.

**Conclusions:**

A two-step process toward the chiral furfuryl alcohol was successfully developed by integrating chemical catalysis with enzyme catalysis, with excellent enantioselectivities. This work demonstrates the power of the combination of chemo- and biocatalysis for selective valorization of biobased furans.

**Graphic abstract:**

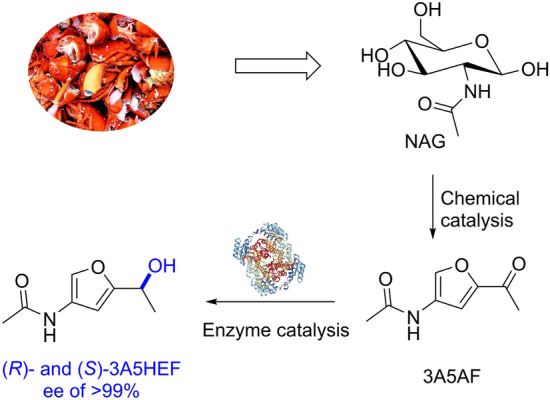

**Supplementary Information:**

The online version contains supplementary material available at 10.1186/s40643-021-00435-w.

## Introduction

Nitrogen-containing chemicals constitute a group of valuable substances that have found wide applications in pharmaceutical, agricultural chemical, polymer, chemical, and food industries. It has been reported that most of the best-selling drugs as well as all the top agrochemicals are N-containing chemicals (Chen et al. 2016, 2020, 2021). In addition, the widespread polyimide and polyamide nylon materials are derived from N-containing monomers, with the market of  >  28 billion USD in 2019 (Chen et al. 2021). Currently, most of industrial N-containing chemicals are obtained by introducing the nitrogen element from NH_3_ and its derivatives into fossil-based intermediates via chemical transformations. In general, NH_3_ is produced by the energy-extensive Haber process (Erisman et al. 2008; Pham et al. 2020). The sustained prevalence of N-containing groups in many important chemicals has greatly driven the development of efficient and sustainable catalytic routes toward organonitrogen chemicals.

Recently, the production of biobased chemicals from renewable and carbon–neutral biomass has attracted considerable interest (Tuck et al. 2012; Sheldon 2014, 2018), due to the gradual depletion of petroleum resources and increased environmental issues (e.g., global warming). Two routes were established to produce biobased N-containing chemicals. One route is that biomass materials such as cellulose and lignin serve as renewable carbon sources to produce the intermediates, followed by incorporating N-functional groups using NH_3_, thus affording N-containing chemicals (He et al. 2020). The other one is to directly synthesize N-containing chemicals from N-containing biomass feedstocks such as chitin and chitosan (Chen et al. 2021; Dai et al. 2020; Hülsey et al. 2018; Shi et al. 2021), which is free of use of external NH_3_ and thus leads to significantly reduced energy consumption. Currently, utilization of N-containing biopolymers as raw materials to produce chemicals and materials received less attention compared to cellulose and lignin. In fact, chitin is the second most abundant biopolymer after cellulose on earth, with the annual production of approximately 100 billion tons (Hülsey et al. 2018). In addition, there is around 7 wt% nitrogen in chitin; hence, it is a promising feedstock for the production of N-containing chemicals.

**Scheme 1:**
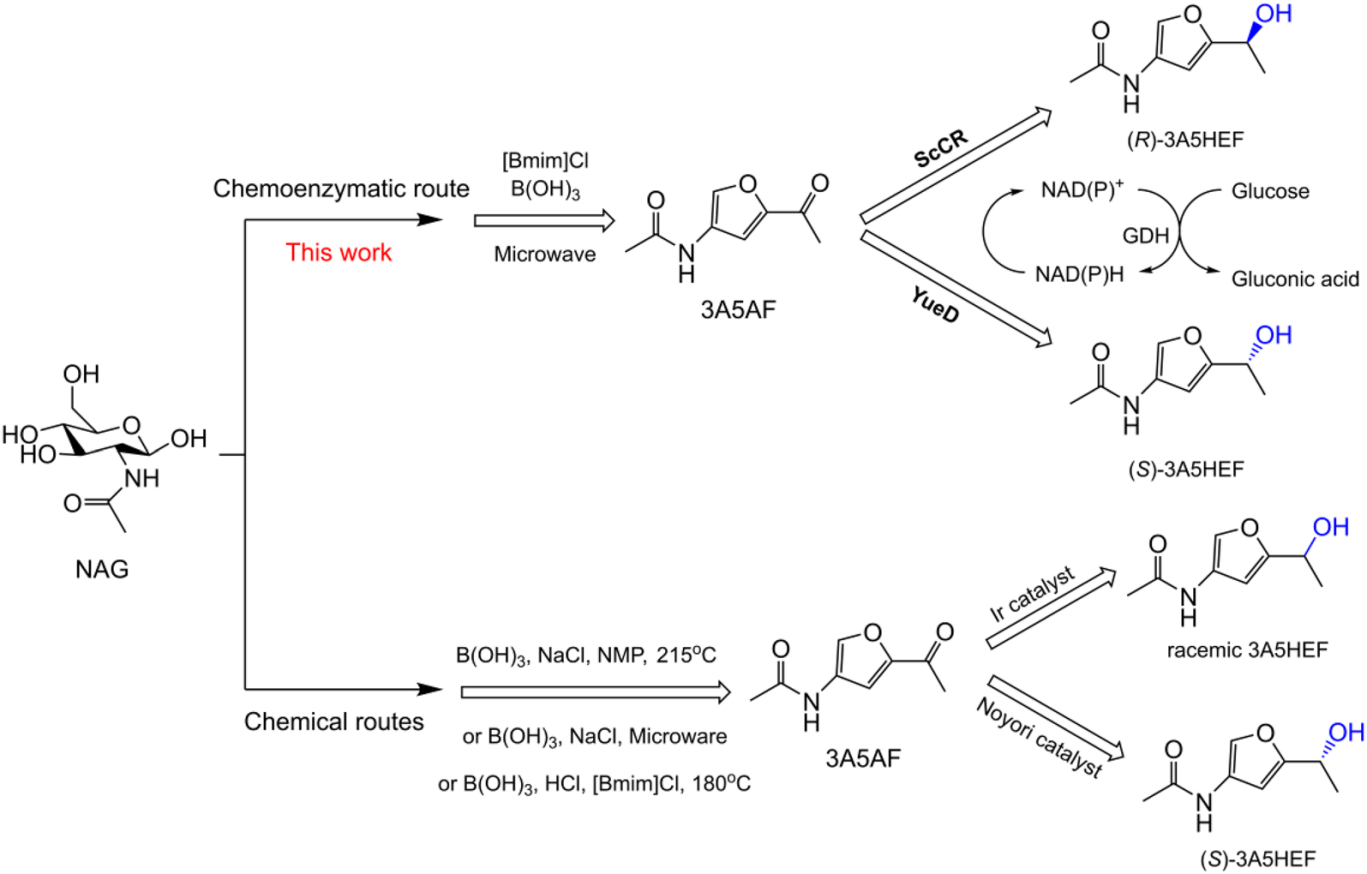
Chemical and chemoenzymatic routes toward chiral 3A5HEF from NAG

3-Acetamido-5-acetylfuran (3A5AF) that can be obtained from N-acetyl-D-glucosamine (NAG) and chitin is a versatile N-containing furan platform chemical. Sperry and co-workers reported the synthesis of an anticancer alkaloid proximicin A and a dihydrodifuropyridine scaffold from 3A5AF (Pham et al. 2018; Sadiq et al. 2018). Recently, this chemical was converted to a group of structurally diverse N-scaffolds such as aminated bicyclic ethers, bicyclic pyrrolidines and pyridines via multi-step chemical transformations (Pham et al. 2020, 2019a, b). 3-Acetamido-5-(1-hydroxylethyl)furan (3A5HEF), a chiral furfuryl alcohol derived from 3A5AF, is the precursor for the synthesis of the rare 2-amino sugar L-rednose, which constitutes the key structural unit of many pharmacologically active natural products such as rudolphomycin (Doyle et al. 1979), anthracycline CG21-C (Johdo et al. 1996), and saquayamycins (Shaaban et al. 2012). Kerton and co-workers described the reduction of 3A5AF to 3A5HEF using stoichiometric NaBH_4_ or via transfer hydrogenation using an Ir-based catalyst (Scheme 1); although good yields were obtained, no enantioselectivity was reported (Liu et al. 2017). Sperry and co-workers reported the synthesis of (*S*)-3A5HEF from 3A5AF in the presence of high-pressure H_2_ using an Ru-based Noyori catalyst (Scheme 1); after 7 d, the desired product was obtained with a yield of 91% and an enantiomeric excess (ee) value of 91% (Pham et al. 2019c). Therefore, it remains a great challenge to develop an efficient and sustainable catalytic protocol to produce this N-containing chiral furfuryl alcohol in enantiopure form. In terms of selectivity and environmental friendliness, biocatalysts appear to excel the chemical counterparts for organic transformations (Sheldon and Woodley 2018). Therefore, we envisage that selective and green synthesis of enantiopure chiral 3A5HEF may be implemented using alcohol dehydrogenases (ADHs), also known as carbonyl reductases (CRs), since asymmetric reduction of prochiral ketones by these enzymes has been established well (Ni and Xu 2012; Goldberg et al. 2007; Hollmann et al. 2021; An et al. 2019). In this work, we report a new chemoenzymatic reaction that combines ionic liquid/boric acid that catalyze the dehydration of NAG with CRs that asymmetrically reduce 3A5AF for producing enantiopure (*R*)- and (*S*)-3A5HEF (Scheme 1). Enzymatic asymmetric reduction of 3A5AF was optimized. The desired products were obtained with good yields and excellent enantioselectivities. In addition, the enzymatic synthesis of (*R*)-3A5HEF was scaled up.

## Materials and methods

### Biological and chemical materials

NAG (97%), 1-butyl-3-methylimidazolium chloride [(Bmim)Cl, 97%], dimethyl sulfoxide-*d*_6_ (DMSO-*d*_6_, 99.9%), ethyl 4-chloro-3-oxobutanoate (COBE, 95%), benzaldehyde (98.5%), and β-nicotinamide adenine dinucleotide (NADH, 98%) were purchased from Macklin Biochemical Co., Ltd. (Shanghai, China). Boric acid [B(OH)_3_ 99.5%] was obtained from Qiangsheng Functional Chemical Co., Ltd. (Jiangsu, China). β-Nicotinamide adenine dinucleotide phosphate (NADPH, 98%) was obtained from Yuanye Biotechnology Co., Ltd. (Shanghai, China). The recombinant plasmids harboring ADH434 and AAD1669 from *Meyerozyma guilliermondii* SC1103, CR from *Acetobacter* sp. CCTCC M209061 (AceCR), ADH from *Synechocystis* sp. (SynADH, GenBank Accession Number CP003265.1) and horse liver ADH (HLADH, Gene ID: 100034242) were maintained in our laboratory. The plasmids harboring CR from *Bacillus* sp. ECU0013 (YueD) and CR from *Streptomyces coelicolor* (ScCR, GenBank accession number NC003888.1) were kindly donated by Prof. Hui-Lei Yu and Prof. Gao-Wei Zheng of East China University of Science and Technology, respectively. The gene of ADH from *Ralstonia* sp. DSM 6428 (RalADH, GenBank accession number EU485985) was synthesized by GenScript Biotechnology Co., Ltd. (Nanjing, China).

### 3A5AF preparation

The preparation of 3A5AF was based on a previous method with some modifications (Omari et al. 2012). Briefly, 10 g NAG, 5.59 g B(OH)_3_, 75 g [Bmim]Cl were added to a 150 mL three-necked flask and mixed. The mixture was heated under microwave irradiation at 180 °C for 10 min. After cooling to room temperature, the reaction mixture was diluted with deionized water to 500 mL and mixed for 1 min. Extraction was performed with 3  ×  500 mL ethyl acetate. The combined organic phases were evaporated under vacuum at 50 °C. The resulting residue was subjected to flash column chromatograph [petroleum ether/ethyl acetate (11/10, v/v), the retention factor (R_f_) of 0.21], according to a previous method (Drover et al. 2012). Its structure was characterized by NMR. ^1^H and ^13^C NMR spectra were recorded using a 600 MHz Bruker NMR (AVANCE III HD 600, Bruker, Switzerland).

### Chemical synthesis of racemic 3A5HEF using NaBH_***4***_

First, 189 mg (1.13 mmol) of 3A5AF was dissolved in 10 mL of anhydrous ethanol. Next, 127.2 mg (3.36 mmol) of NaBH_4_ was dissolved in 10 mL anhydrous ethanol under N_2_ flow. With stirring, the NaBH_4_ solution was added dropwise to the 3A5AF solution at 4 °C, followed by adding 10 mL anhydrous ethanol. After 1 h, the reaction mixture was stirred at room temperature overnight. Upon reaction, the mixture was filtered, and the filtrate was concentrated under vacuum at 40 °C. The residue was dissolved in 30 mL deionized water, followed by extraction with 4  ×  50 mL ethyl acetate. The combined organic phases were evaporated under vacuum at 50 °C, affording yellow oil. Its structure and purity were characterized by NMR, and HPLC, respectively.

### Expression and purification of ScCR and YueD

The recombinant cells [*Escherichia coli* BL21(DE3)/pET28a-ScCR and *E. coli* BL21(DE3)/pET28a-YueD] were inoculated on 100 μg/mL kanamycin-containing Luria–Bertani (LB) agar plates. A single colony was picked out and pre-cultivated in 20 mL kanamycin (100 μg/mL)-containing LB medium at 37 °C and 160 r/min for approximately12 h. Then, 1% seed culture was inoculated into 100 mL LB medium and incubated at 37 °C and 160 r/min. When the optical density at 600 nm (OD_600_) of the culture reached 0.6–0.8, isopropy-β-D-thiogalactoside (IPTG) was added at a final concentration of 0.5 mM to induce the enzyme expression at 20 °C and 160 r/min for 20 h. Then, the cells were harvested by centrifugation (5200×*g*, 10 min) at 4 °C, washed twice with 0.9% NaCl solution, and resuspended in sodium phosphate buffer (0.1 M, pH 7.0). Besides, the recombinant plasmid pET28a_GDH_ScCR was constructed (Additional file 1: Scheme S1) and was transformed into the competent cells of *E. coli* BL21(DE3), and the protein expression was conducted under the above-mentioned conditions.

The harvested cells were resuspended in binding buffer (pH 7.0, 0.1 M sodium phosphate buffer, 0.5 M NaCl and 0.05 M imidazole) and disrupted by sonication for 15 min (30% power, 3 s on, 5 s off and at 4 °C). The cell lysate was centrifuged at 4 °C (15,285×*g*) for 10 min, and the supernatant was loaded onto a HisTrap FF crude column (GE, USA) equilibrated with binding buffer. Then, the samples were eluted with buffer (pH 7.0, 0.1 M sodium phosphate buffer, 0.5 M NaCl and 0.1 M imidazole) in a flow rate of 2 mL/min to remove the impure proteins. Next, the elution of the target enzymes was conducted with an elution buffer (pH 7.0, 0.1 M sodium phosphate buffer, 0.5 M NaCl and 0.4 M imidazole). The fractions containing target enzymes were desalted by HisTrap™ Desalting column (5 mL) with desalting buffer (pH 7.0, 0.1 M sodium phosphate buffer). The purified protein fractions were subjected to SDS-PAGE analysis to indicate its purity (Additional file 1: Figure S1). In addition, the protein concentrations were measured by the Bradford protocol (Yang et al. 2021).

### General procedure for biocatalytic reduction of 3A5AF

Typically, the purified enzyme (1 mg/mL) was dissolved in 1 mL of sodium phosphate buffer (0.1 M, pH 7.0) containing 10 mM 3A5AF and 5% DMSO (v/v). Upon the addition of NAD(P)H (0.1 mM), glucose (20 mM) and GDH cell-free extract (0.05 mg/mL), the reaction mixtures were incubated at 35 °C and 150 r/min. Next, aliquots were withdrawn from the reaction mixtures at specified time intervals and diluted with the corresponding mobile phase prior to high-performance liquid chromatography (HPLC) analysis. For determining the product ee, samples were extracted twice with equivalent volume of ethyl acetate, and the organic phase was subjected to chiral HPLC analysis. The initial reaction rate was defined as the formed products concentration at the initial stage (within 0.5 h). The conversion was defined as the ratio of the consumed substrate to the initial substrate amount (in mol). Likewise, the yield was defined as the ratio of the formed product amount to the theoretical value based on the initial substrate amount (in mol). All the experiments were conducted in duplicate, and the values were expressed as the means  ±  standard deviations.

### Scale-up synthesis and preparation of (*R*)-3A5HEF

ScCR (80 mg) was added into 20 mL of sodium phosphate buffer (0.1 M, pH 7.0) containing 0.6 mmol 3A5AF, 0.1 mM NADH, 60 mM glucose, 0.6 mmol CaCO_3_, 0.05 mg/mL GDH cell-free extract, 10% DMSO (v/v). The reaction mixture was incubated at 35 °C and 150 r/min for 48 h. After enzyme inactivation by heating, the solution was extracted 4 times with equal volume of ethyl acetate. The organic phases were combined and dried with anhydrous Na_2_SO_4_ overnight. Upon removal of organic solvent, the residual was subjected to flash column chromatography [petroleum ether/ethyl acetate (1/2, v/v), the retention factor (R_f_) of 0.2], affording the purified product.

### Analytical methods

The reaction mixtures were analyzed on a Zorbax Eclipse XDB-C18 column (4.6 mm × 250 mm, 5 μm, Agilent, USA) using a reversed phase HPLC (Waters, USA) equipped with a Waters 1525 pump and a 2489 UV detector. The mixture of acetonitrile/0.4% (NH_4_)_2_SO_4_ solution with pH 3.5 (20/80, v/v) was used as the mobile phase with the flow rate of 0.6 mL/min. The retention times of 3A5AF (228 nm) and 3A5HEF (210 nm) were 9.8 min and 6.7 min, respectively. In addition, the product ee values were determined by HPLC analysis using an OJ-H Chiral column (4.6  ×  250 mm; Daicel Co., Japan). The mobile phase is *n*-hexane/isopropanol (84/16, v/v) with a flow rate of 0.7 mL/min. The retention times of (*R*)-3A5HEF and (*S*)-3A5HEF were 19.4 and 17.8 min, respectively. The specific rotation values of chiral furfuryl alcohols (Additional file 1: Table S1) were determined via an automatic polarimeter P810 (Hanon Instruments, Inc., China). To assay specific rotation values of 3A5HEF at 589 nm and 25 °C in ethanol, a concentration of 17.9 mg/mL was used. Absolute configurations of the products were based on comparison of their specific optical rotations with those in the literature.

## Results and discussion

### Enzyme screening

The chemically catalytic synthesis of 3A5AF was performed by combining ionic liquid [Bmim]Cl with boric acid under microwave irradiation, according to a previous method (Drover et al. 2012). Upon solvent extraction and column chromatography, the desired product was obtained with an isolated yield of approximately 31%.

To date, there are few reports on biocatalytic asymmetric synthesis of chiral furfuryl alcohols in the literature (Blume et al. 2016). Therefore, six CRs/ADHs were tested for asymmetric reduction of 3A5AF (Table 1), based on their good catalytic performances in the reduction of prochiral ketones (Wang et al. 2011; Ni et al. 2011; Nealon et al. 2015; Wei et al. 2017; Lavandera et al. 2008; Vidal et al. 2009). It was found that only ScCR and YueD showed good catalytic activities toward 3A5AF (entries 1–2). ScCR provided (*R*)-alcohol with the yield of  >  99% and ee of  >  99% in 9 h (entry 1), while (*S*)-product was obtained using YueD, with a good yield and an excellent ee (entry 2). 3A5AF seemed not to be a good substrate of RalADH, AceCR and SynADH, since low yields (6–12%) were achieved after a long reaction period (entries 3–5). Although HLADH was reported to have broad substrate specificity (Nealon et al. 2015), it could not accept 3A5AF as a substrate (entry 6). In addition, both ADH434 and AAD1669 from *M. guilliermondii* SC1103 that proved to be good catalysts for the reduction of 5-hydroxymethylfurfural (HMF) (Xia et al. 2020), an analog of 3A5AF, were unable to reduce 3A5AF as well (data not shown), likely due to great steric hindrance of this substrate.

**Table 1:** Asymmetric synthesis of 3A5HEF catalyzed by CRs/ADHs

Entry	Enzyme	Time (*h*)	Product yield (%)	Product ee (%)
1	ScCR	9	> 99	> 99 (R)
2	YueD^a^	9	90	> 99 (S)
3	RalADH^a^	60	12	> 99 (S)
4	AceCR	60	8	> 99 (R)
5	SynADH^a^	60	6	> 99 (R)
6	HLADH	60	na^b^	na

Reaction conditions: 10 mM 3A5AF, 1 mg/mL purified enzyme, 0.05 mg/mL GDH cell-free extract, 20 mM glucose, 0.1 mM NADH, 1 mL sodium phosphate buffer (0.1 M, pH 7) containing 5% (v/v) DMSO, 30 °C, 150 r/min

^a^NADPH used

^b^No activity

### Whole-cell catalysis

**Fig. 1:**
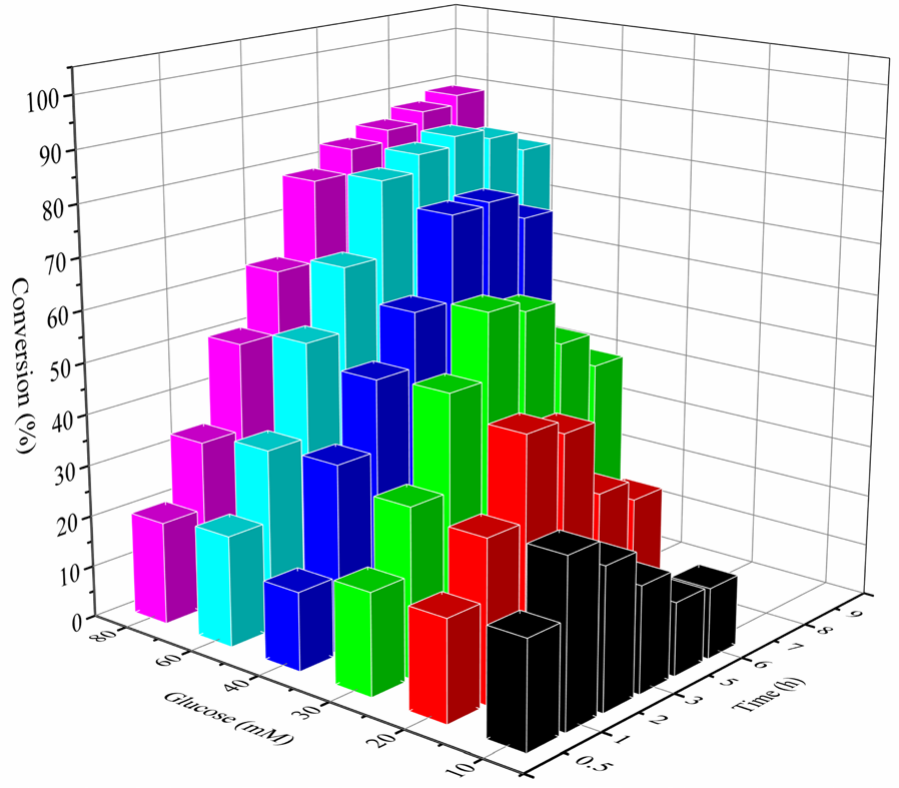
Effect of glucose concentrations on asymmetric reduction of 3A5AF catalyzed by recombinant *E.coli*_GDH_ScCR cells. Reaction conditions: 10 mM 3A5AF, 20 mg/mL *E.coli*_GDH_ScCR, 10–80 mM glucose, 1 mL sodium phosphate buffer (0.1 M, pH 7.0) containing 5% DMSO (v/v), 30 °C, 150 r/min

From an economic perspective, whole-cell catalysts appear be preferred to the isolated enzymes for biotransformations, since whole-cell catalysts can be more readily and inexpensively prepared. Besides, whole-cell catalysts appear to be more stable, due to the protection by cell membrane and/or cell wall from enzyme inactivation caused by potentially harmful surroundings (Carballeira et al. 2009). The processes involving cofactor regeneration can be significantly simplified using whole-cell catalysts (Wachtmeister and Rother 2016). Therefore, whole-cell biocatalytic reduction of 3A5AF was performed. First, a substrate-coupled NADH regeneration method was applied to synthesize (*R*)-3A5HEF by whole cells expressing ScCR (Additional file 1: Figure S2). It was found that much high molar ratios of co-substrate (2-propanol) to 3A5AF (up to 50:1) were required to derive the synthesis of this chiral furfuryl alcohol, due to the thermodynamic equilibrium issue (Itoh et al. 2002; Hummel and Gröger 2014). The maximal substrate conversion of approximately 70% was obtained (Additional file 1: Figure S2). Then, an enzyme-coupled NADH recycling system was constructed by co-expressing glucose dehydrogenase (GDH) with ScCR in *E. coli* (Additional file 1: Scheme S1), and whole-cell biocatalytic reduction was performed (Fig. 1). Although the molar ratios of co-substrate (glucose) to 3A5AF (about 6–8:1) were significantly reduced, the concentrations of glucose used remain high, likely due to oxidative assimilation of glucose by *E. coli* cells. It would impair the sustainability of this process and also have a negative effect on the downstream product purification, especially at high substrate concentrations. To reduce glucose consumption, therefore, enzyme-catalyzed asymmetric reduction of 3A5AF using ScCR coupled with GDH was performed in the subsequent studies.

### Process optimization

**Fig. 2:**
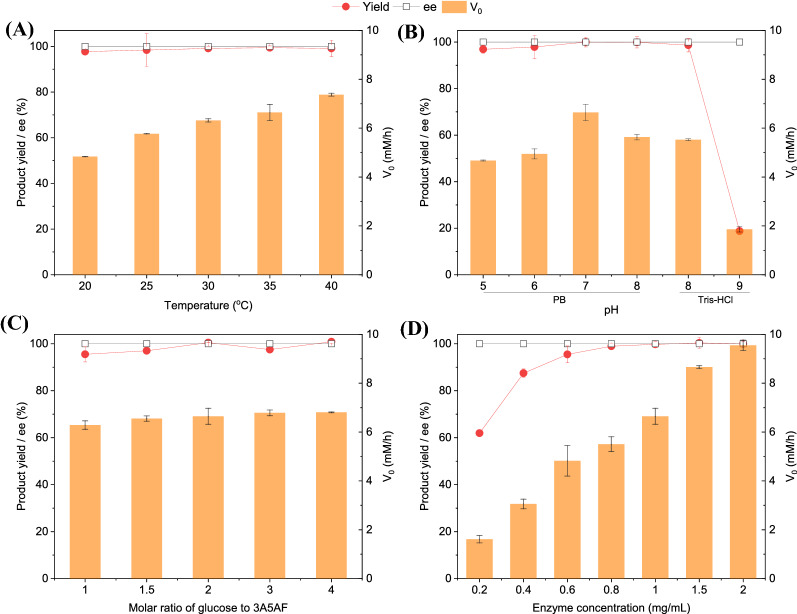
Effects of temperature (**A**), pH (**B**), substrate molar ratios (**C**), and ScCR concentrations (**D**) on enzymatic synthesis of (*R*)-3A5HEF. General reaction conditions: 10 mM 3A5AF, 1 mg/mL ScCR, 0.05 mg/mL GDH cell-free extract, Mol_glucose_: Mol_3A5AF_  =  2:1, 0.1 mM NADH, 1 mL sodium phosphate buffer (0.1 M, pH 7) containing 5% DMSO (v/v), 35 °C, 150 r/min, 7 h; **A** 20–40 °C; **B** pH 5–9; **C** 1–4; **D** 0.2–2 mg/mL

Figure 2 shows the effect of four key parameters on the enzymatic synthesis of (*R*)-3A5HEF. The effect of temperature on biocatalytic synthesis of (*R*)-3A5HEF is depicted in Fig. 2A. It was found that the initial reaction rates (V_0_) greatly increased from 4.8 to 7.4 mM/h with the increment of temperature, although the product yields were comparable in all cases. It was reported that the half-live of ScCR was around 169 h at 30 °C, while it was markedly reduced to 81 h at 40 °C (Wang et al. 2011). Considering the thermostability of ScCR, the following studies were conducted at 35 °C. As shown in Fig. 2B, the enzyme ScCR displayed the highest activity (approximately 6.6 mM/h) in the reduction of 3A5AF at pH 7, which is close to its optimal pH (pH 6.5) (Wang et al. 2011). In addition, good catalytic activities were observed within a broad pH range (pH 5–8), indicating the great application potential of this enzyme.

Herein glucose served as the sacrificial substrate for enzymatic regeneration of NADH. Therefore, the effect of the substrate molar ratios (glucose/3A5AF) on the enzymatic synthesis of 3A5HEF was studied (Fig. 2C). The initial reaction rates as well as the product yields were comparable when the substrate molar ratios were varied from 1 to 4. In addition, even at the substrate molar ratio of 1, a product yield of 96% and an initial reaction rate of 6.3 mM/h were obtained, which indicates that the enzyme-coupled strategy is much advantageous over the substrate-coupled one in terms of environmental (E) factor (Sheldon 2017). Then, the influence of the ScCR concentrations on the enzymatic synthesis of 3A5HEF was explored (Fig. 2D). It was found that the initial reaction rates significantly increased with the increased enzyme concentrations. The desired product was obtained in the yields of  >  95% within 7 h, with more than 0.6 mg/mL of ScCR.

### Effect of the substrate loadings

**Fig. 3:**
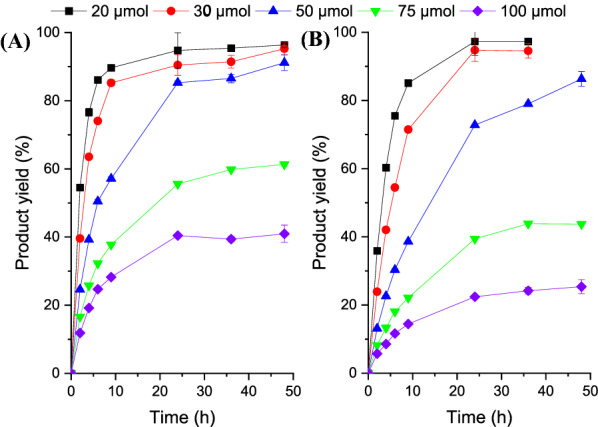
Effect of the substrate loadings on asymmetric synthesis of 3A5HEF by ScCR (**A**) and YueD (**B**). Reaction conditions: 20–100 μmol 3A5AF, 4 mg/mL enzyme, 0.05 mg/mL GDH cell-free extract, 0.1 mM NAD(P)H, 20–100 μmol CaCO_3_, molar ratio of glucose to 3A5AF of 2, 1 mL sodium phosphate buffer (0.1 M, pH 7) containing 10% DMSO (v/v), 35 °C, 150 r/min. For YueD catalysis, sodium phosphate buffer (0.1 M, pH 8) was used

Then, the enzymatic synthesis of (*R*)- and (*S*)-3A5HEF was performed by ScCR and YueD, respectively, at higher substrate loadings (Fig. 3). Notably, 3A5AF is a poorly aqueous soluble chemical; its solubility is approximately 10 and 11 mM in 5% and 10% (v/v) DMSO solutions at 35 °C, respectively. However, the product 3A5HEF is highly soluble in 10% (v/v) DMSO solution, with the solubility of  >  1 M. Therefore, most of substrate was suspended in aqueous solutions at the substrate loadings of  >  20 μmol in 1 mL solvent. It was interestingly observed that 3A5AF could be smoothly reduced into the desired product by the two enzymes at the substrate loadings of 20–50 μmol. With the substrate loading of 50 μmol, for example, (*R*)-3A5HEF was obtained with a 91% yield after 48 h (Fig. 3A). When the substrate loading increased to 75 μmol, a moderate yield (approximately 60%) was achieved, and even the yield was lower at 100 μmol 3A5AF. To uncover the underlying reasons, the inhibition effects of substrate and product on the enzyme were studied (Additional file 1: Figure S4), since many alcohol dehydrogenases were reported to suffer from such effects (Yang et al. 2020; Wratten and Cleland 1963). No substrate inhibition was observed within substrate concentration range of 1–10 mM (Additional file 1: Figure S4A). However, a great product inhibition effect on the enzyme activity was observed in the presence of more than 60 mM of 3A5HEF (Additional file 1: Figure S4B). The low yields in the cases of 75–100 mM may be partially attributed to great product inhibition. In addition, (*S*)-3A5HEF was synthesized with good yields (> 86%) using YueD when the substrate loadings were less than 50 μmol (Fig. 3B). At higher substrate loadings, much lower yields (25–44%) were obtained. Based on the kinetic studies (Additional file 1: Table S3), ScCR showed lower substrate affinity toward 3A5AF than YueD (*K*_m_, 6.3 mM vs 0.5 mM); however, the *k*_cat_ value of the former was much higher than that of the latter (0.1 vs 0.0007 s^−1^), thus resulting in the higher catalytic efficiency of ScCR.

To demonstrate practical applicability of this enzymatic route, the synthesis of (*R*)-3A5HEF was conducted on a preparative scale (20 mL). The desired product was synthesized with a yield of 93% after 48 h, based on HPLC analysis. Upon solvent extraction and column chromatograph, (*R*)-3A5HEF was obtained with an isolated yield of 65% and ee of  >  99%. Its purity is approximately 95%, based on HPLC analysis (Additional file 1: Figure S5). Compared to the metal-mediated process (Pham et al. 2019c), this biocatalytic route shows many advantages such as high efficiency, excellent enantioselectivity, mild reaction conditions and environmental friendliness.

## Conclusions

In summary, a novel chemoenzymatic approach toward enantiopure N-containing chiral furfuryl alcohols from biomass-derived NAG was successfully developed in this work, with the isolation of 3A5AF. The carbonyl reductases ScCR and YueD were capable of selective reduction of 3A5AF to (*R*)- and (*S*)-5A5HEF, respectively, with the yields of up to  >  99% and ee of  >  99%. From the sustainable chemistry point of view, the enzyme-coupled strategy (glucose/GDH) for recycling reduced nicotinamide cofactor seems to excel the substrate-coupled one (2-propanol). A good yield could be obtained in enzymatic reduction of 3A5AF in the presence of one equivalent of glucose. In addition, the scale-up synthesis of (*R*)-5A5HEF was demonstrated. In spite of good results, some issues (poor water-solubility of substrate) accompanied with this biocatalytic route should still be addressed by reaction engineering strategies in the future. For instance, the actual substrate concentrations may be increased significantly using organic solvent/water biphasic systems, thus resulting in the improved catalytic efficiency. In addition, the application of the enzyme immobilization or whole-cell biocatalysts coexpressing formate dehydrogenase and CRs may lead to the greatly reduced cost of the enzymes for its future large-scale applications.

### Supplementary Information

**Additional file 1: Figure S1.** SDS-PAGE analysis of purified ScCR and YueD. **Figure S2. **Effect of 2-propanol concentrations on asymmetric reduction of 3A5AF catalyzed by *E. coli*_ScCR cells. **Figure S3. **SDS-PAGE analysis of protein present in the supernatant and precipitant of *E. coli*_GDH_ScCR. **Figure S4. **Effect of substrate concentrations (A) and product concentrations (B) on ScCR-catalyzed synthesis of 3A5HEF. **Figure S5. **HPLC analysis of (*R*)-3A5HEF obtained on a preparative-scale experiment. **Figure S6.** SDS-PAGE analysis of purified RalADH. **Figure S7.** SDS-PAGE analysis of purified AceCR. **Figure S8.** SDS-PAGE analysis of purified SynADH. **Figure S9.** SDS-PAGE analysis of purified HLADH. Lane M: protein marker. **Figure S10.** SDS-PAGE analysis of crude ADH434 and AAD1669. **Figure S11. **^1^H NMR of 3A5AF (DMSO-*d*6, 600 MHz). **Figure S12. **^13^C NMR of 3A5AF (DMSO-*d*6, 125 MHz). **Figure S13. **^1^H NMR of 3A5HEF (DMSO-*d*6, 600 MHz). **Figure S14. **^13^C NMR of 3A5HEF (DMSO-*d*6, 125 MHz). **Figure S15. **The chiral HPLC spectrum of 3A5HEF prepared by chemical method. **Figure S16. **The chiral HPLC spectrum of (*R*)-3A5HEF obtained on a preparative-scale experiment. **Figure S17. **The chiral HPLC spectrum of (*S*)-3A5HEF. **Figure S18. **HPLC analysis of the reaction mixture in enzymatic reduction of 3A5AF. **Figure S19. **The chiral HPLC spectrum of the reaction mixture in enzymatic reduction of 3A5AF to (*R*)-3A5HEF. **Figure S20. **The chiral HPLC spectrum of the reaction mixture in enzymatic reduction of 3A5AF to (*S*)-3A5HEF. **Table S1. **Specific optical rotations of the chiral furfuryl alcohols. **Table S2. **Specific activities of various CRs/ADHs. **Table S3. **Kinetic parameters of two enzymes using 3A5AF as a substrate.

## Data Availability

They are included within the article and its Additional file 1.

## References

[CR1] An J, Nie Y, Xu Y (2019). Structural insights into alcohol dehydrogenases catalyzing asymmetric reductions. Crit Rev Biotechnol.

[CR2] Blume F, Liu Y-C, Thiel D, Deska J (2016). Chemoenzymatic total synthesis of (+)- and (−)-cis-osmundalactone. J Mol Catal B Enzym.

[CR3] Carballeira JD, Quezada MA, Hoyos P, Simeo Y, Hernaiz MJ, Alcantara AR, Sinisterra JV (2009). Microbial cells as catalysts for stereoselective red-ox reactions. Biotechnol Adv.

[CR4] Chen X, Yang H, Yan N (2016). Shell biorefinery: dream or reality?. Chem Eur J.

[CR5] Chen X, Liu Y, Wang J (2020). Lignocellulosic biomass upgrading into valuable nitrogen-containing compounds by heterogeneous catalysts. Ind Eng Chem Res.

[CR6] Chen X, Song S, Li H, Gözaydın G, Yan N (2021). Expanding the boundary of biorefinery: organonitrogen chemicals from biomass. Acc Chem Res.

[CR7] Dai J, Li F, Fu X (2020). Towards shell biorefinery: advances in chemical-catalytic conversion of chitin biomass to organonitrogen chemicals. Chemsuschem.

[CR8] Doyle TW, Nettleton DE, Grulich RE, Balitz DM, Johnson DL, Vulcano AL (1979). Antitumor agents from the bohemic acid complex. 1 4. Structures of rudolphomycin, mimimycin, collinemycin, and alcindoromycin. J Am Chem Soc.

[CR9] Drover MW, Omari KW, Murphy JN, Kerton FM (2012). Formation of a renewable amide, 3-acetamido-5-acetylfuran, via direct conversion of N-acetyl-D-glucosamine. RSC Adv.

[CR10] Erisman JW, Sutton MA, Galloway J, Klimont Z, Winiwarter W (2008). How a century of ammonia synthesis changed the world. Nat Geosci.

[CR11] Goldberg K, Schroer K, Lütz S, Liese A (2007). Biocatalytic ketone reduction—a powerful tool for the production of chiral alcohols—part I: processes with isolated enzymes. Appl Microbiol Biotechnol.

[CR12] He J, Chen L, Liu S, Song K, Yang S, Riisager A (2020). Sustainable access to renewable N-containing chemicals from reductive amination of biomass-derived platform compounds. Green Chem.

[CR13] Hollmann F, Opperman DJ, Paul CE (2021). Biocatalytic reduction reactions from a chemist’s perspective. Angew Chem Int Ed.

[CR14] Hülsey MJ, Yang H, Yan N (2018). Sustainable routes for the synthesis of renewable heteroatom-containing chemicals. ACS Sustain Chem Eng.

[CR15] Hummel W, Gröger H (2014). Strategies for regeneration of nicotinamide coenzymes emphasizing self-sufficient closed-loop recycling systems. J Biotechnol.

[CR16] Itoh N, Matsuda M, Mabuchi M, Dairi T, Wang J (2002). Chiral alcohol production by NADH-dependent phenylacetaldehyde reductase coupled with in situ regeneration of NADH. Eur J Biochem.

[CR17] Johdo O, Yoshioka T, Naganawa H, Takeuchi T, Yoshimoto A (1996). New betaclamycin and aclarubicin analogs obtained by prolonged microbial conversion with an aclarubicin-negative mutant. J Antibiot.

[CR18] Lavandera I, Kern A, Ferreira-Silva B, Glieder A, de Wildeman S, Kroutil W (2008). Stereoselective bioreduction of bulky-bulky ketones by a novel ADH from *Ralstonia* sp.. J Org Chem.

[CR19] Liu Y, Stähler C, Murphy JN, Furlong BJ, Kerton FM (2017). Formation of a renewable amine and an alcohol via transformations of 3-acetamido-5-acetylfuran. ACS Sustain Chem Eng.

[CR20] Nealon CM, Musa MM, Patel JM, Phillips RS (2015). Controlling substrate specificity and stereospecificity of alcohol dehydrogenases. ACS Catal.

[CR21] Ni Y, Xu J-H (2012). Biocatalytic ketone reduction: a green and efficient access to enantiopure alcohols. Biotechnol Adv.

[CR22] Ni Y, Li C-X, Wang L-J, Zhang J, Xu J-H (2011). Highly stereoselective reduction of prochiral ketones by a bacterial reductase coupled with cofactor regeneration. Org Biomol Chem.

[CR23] Omari KW, Dodot L, Kerton FM (2012). A simple one-pot dehydration process to convert n-acetyl-d-glucosamine into a nitrogen-containing compound, 3-acetamido-5-acetylfuran. Chemsuschem.

[CR24] Pham TT, Chen X, Yan N, Sperry J (2018). A novel dihydrodifuropyridine scaffold derived from ketones and the chitin-derived heterocycle 3-acetamido-5-acetylfuran. Monatsh Chem.

[CR25] Pham TT, Lindsay AC, Kim S-W, Persello L, Chen X, Yan N, Sperry J (2019). Two-step preparation of diverse 3-amidofurans from chitin. ChemistrySelect.

[CR26] Pham TT, Lindsay AC, Chen X, Gözaydin G, Yan N, Sperry J (2019). Transferring the biorenewable nitrogen present in chitin to several N-functional groups. Sustain Chem Pharm.

[CR27] Pham TT, Gözaydın G, Söhnel T, Yan N, Sperry J (2019). Oxidative ring-expansion of a chitin-derived platform enables access to unexplored 2-amino sugar chemical space. Eur J Org Chem.

[CR28] Pham TT, Chen X, Söhnel T, Yan N, Sperry J (2020). Haber-independent, diversity-oriented synthesis of nitrogen compounds from biorenewable chitin. Green Chem.

[CR29] Sadiq AD, Chen X, Yan N, Sperry J (2018). Towards the shell biorefinery: sustainable synthesis of the anticancer alkaloid proximicin A from chitin. Chemsuschem.

[CR30] Shaaban KA, Ahmed TA, Leggas M, Rohr J (2012). Saquayamycins G-K, cytotoxic angucyclines from *Streptomyces* sp. including two analogues bearing the aminosugar rednose. J Nat Prod.

[CR31] Sheldon RA (2014). Green and sustainable manufacture of chemicals from biomass: state of the art. Green Chem.

[CR32] Sheldon RA (2017). The E factor 25 years on: the rise of green chemistry and sustainability. Green Chem.

[CR33] Sheldon RA (2018). Chemicals from renewable biomass: a renaissance in carbohydrate chemistry. Curr Opin Green Sustain Chem.

[CR34] Sheldon RA, Woodley JM (2018). Role of biocatalysis in sustainable chemistry. Chem Rev.

[CR35] Shi X, Ye X, Zhong H, Wang T, Jin F (2021). Sustainable nitrogen-containing chemicals and materials from natural marine resources chitin and microalgae. Mol Catal.

[CR36] Tuck CO, Pérez E, Horváth IT, Sheldon RA, Poliakoff M (2012). Valorization of biomass: deriving more value from waste. Science.

[CR37] Vidal R, López-Maury L, Guerrero MG, Florencio FJ (2009). Characterization of an alcohol dehydrogenase from the cyanobacterium *Synechocystis* sp. strain PCC 6803 that responds to environmental stress conditions via the Hik34-Rre1 two-component system. J Bacteriol.

[CR38] Wachtmeister J, Rother D (2016). Recent advances in whole cell biocatalysis techniques bridging from investigative to industrial scale. Curr Opin Biotechnol.

[CR39] Wang L-J, Li C-X, Ni Y, Zhang J, Liu X, Xu J-H (2011). Highly efficient synthesis of chiral alcohols with a novel NADH-dependent reductase from *Streptomyces coelicolor*. Bioresour Technol.

[CR40] Wei P, Cui Y-H, Zong M-H, Xu P, Zhou J, Lou W-Y (2017). Enzymatic characterization of a recombinant carbonyl reductase from *Acetobacter* sp. CCTCC M209061. Bioresour Bioprocess.

[CR41] Wratten CC, Cleland WW (1963). Product inhibition studies on yeast and liver alcohol dehydrogenases. Biochemistry.

[CR42] Xia Z-H, Zong M-H, Li N (2020). Catalytic synthesis of 2,5-bis(hydroxymethyl)furan from 5-hydroxymethylfurfual by recombinant *Saccharomyces cerevisiae*. Enzyme Microb Technol.

[CR43] Yang Z, Ye W, Xie Y, Liu Q, Chen R, Wang H, Wei D (2020). Efficient asymmetric synthesis of ethyl (s)-4-chloro-3-hydroxybutyrate using alcohol dehydrogenase smadh31 with high tolerance of substrate and product in a monophasic aqueous system. Org Proc Res Dev.

[CR44] Yang Z-Y, Hao Y-C, Hu S-Q, Zong M-H, Chen Q, Li N (2021). Direct reductive amination of biobased furans to n-substituted furfurylamines by engineered reductive aminase. Adv Synth Catal.

