# Supplementary material for: Chemoenzymatic access to enantiopure N-containing furfuryl alcohol from chitin-derived N-acetyl-D-glucosamine
Source: Bioresour Bioprocess. 2021 Aug 27;8(1):80. doi: 10.1186/s40643-021-00435-w (PMC10992857; doi:10.1186/s40643-021-00435-w)
Supplement: Supplementary file 1 — Additional file 1: Figure S1. SDS-PAGE analysis of purified ScCR and YueD. Figure S2. Effect of 2-propanol concentrations on asymmetric reduction of 3A5AF catalyzed by E. coli_ScCR cells. Figure S3. SDS-PAGE analysis of protein present in the supernatant and precipitant of E. coli_GDH_ScCR. Figure S4. Effect of substrate concentrations (A) and product concentrations (B) on ScCR-catalyzed synthesis of 3A5HEF. Figure S5. HPLC analysis of (R)-3A5HEF obtained on a preparative-scale experiment. Figure S6. SDS-PAGE analysis of purified RalADH. Figure S7. SDS-PAGE analysis of purified AceCR. Figure S8. SDS-PAGE analysis of purified SynADH. Figure S9. SDS-PAGE analysis of purified HLADH. Lane M: protein marker. Figure S10. SDS-PAGE analysis of crude ADH434 and AAD1669. Figure S11. 1H NMR of 3A5AF (DMSO-d6, 600 MHz). Figure S12. 13C NMR of 3A5AF (DMSO-d6, 125 MHz). Figure S13. 1H NMR of 3A5HEF (DMSO-d6, 600 MHz). Figure S14. 13C NMR of 3A5HEF (DMSO-d6, 125 MHz). Figure S15. The chiral HPLC spectrum of 3A5HEF prepared by chemical method. Figure S16. The chiral HPLC spectrum of (R)-3A5HEF obtained on a preparative-scale experiment. Figure S17. The chiral HPLC spectrum of (S)-3A5HEF. Figure S18. HPLC analysis of the reaction mixture in enzymatic reduction of 3A5AF. Figure S19. The chiral HPLC spectrum of the reaction mixture in enzymatic reduction of 3A5AF to (R)-3A5HEF. Figure S20. The chiral HPLC spectrum of the reaction mixture in enzymatic reduction of 3A5AF to (S)-3A5HEF. Table S1. Specific optical rotations of the chiral furfuryl alcohols. Table S2. Specific activities of various CRs/ADHs. Table S3. Kinetic parameters of two enzymes using 3A5AF as a substrate. [file 40643_2021_435_MOESM1_ESM.pdf]

Supplementary Information

Chemoenzymatic access to enantiopure N-containing furfuryl alcohol from chitin-derived N-acetyl-D-glucosamine

Cheng Hao,[a] Min-Hua Zong,[a] Zhi-Lin Wang,[b*] Ning Li[a*]

[a] School of Food Science and Engineering, South China University of Technology, 381 Wushan Road, Guangzhou 510640, China

[b] Agro-biological Gene Research Center, Guangdong Academy of Agricultural Sciences, 20 Jinying Road, Guangzhou, 510640, China

* Corresponding authors.

Z.L. Wang, Email: wangzhilin@gdaas.cn

Dr. N. Li, Email: lining@scut.edu.cn

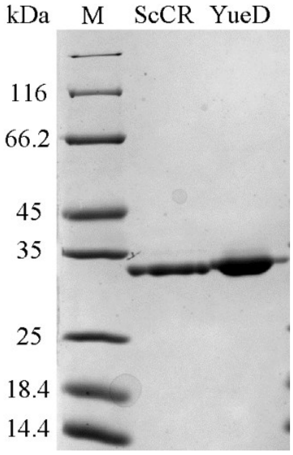

Fig. S1. SDS-PAGE analysis of purified ScCR and YueD. Lane M: protein marker

Table S1. Specific optical rotations of the chiral furfuryl alcohols

Sample	Specific rotation	Used dehydrogenase
(*R*)-3A5HEF	6.7	ScCR
(*S*)-3A5HEF	-6.8	YueD

Conditions: 179 mg of the corresponding sample was added to 10 mL ethanol and measured at 589 nm and 25 °C in 1 dc polarimeter tube.

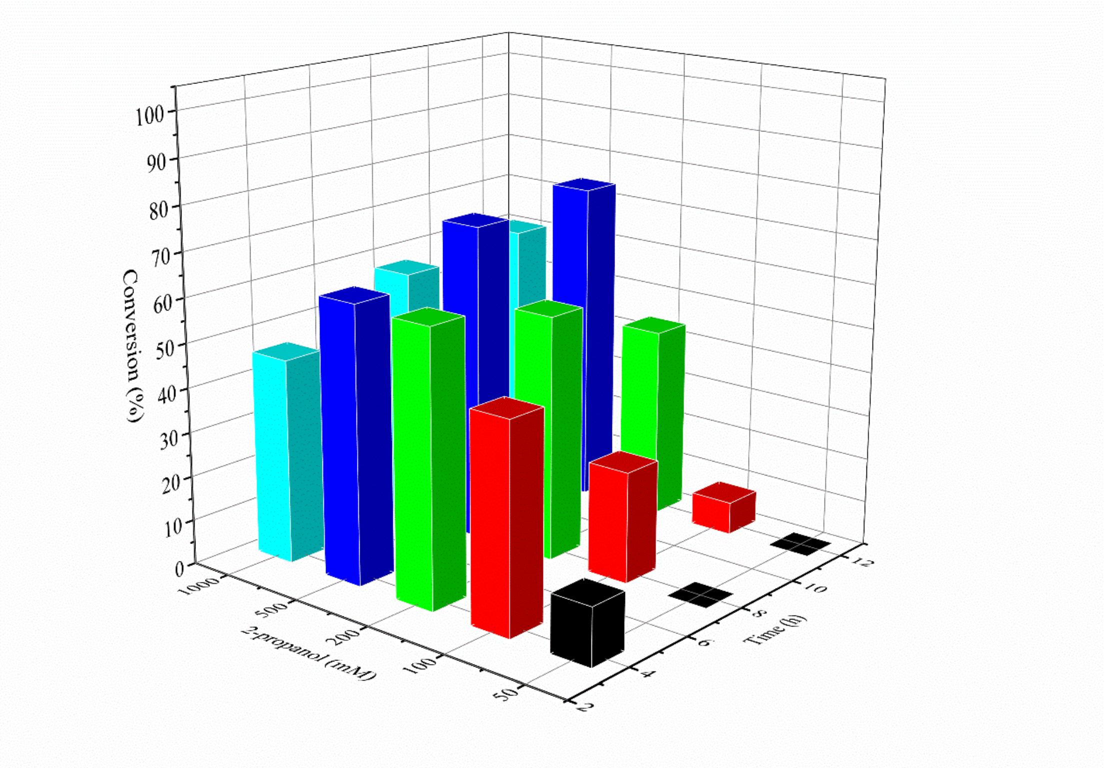

Fig. S2. Effect of 2-propanol concentrations on asymmetric reduction of 3A5AF catalyzed by *E. coli*_ScCR cells. Reaction conditions: 10 mM 3A5AF, 20 mg/mL cells, 50-1000 mM 2-propanol, 1 mL sodium phosphate buffer (0.1 M, pH 7.0) containing 5% DMSO (v/v), 30 °C, 150 r/min.

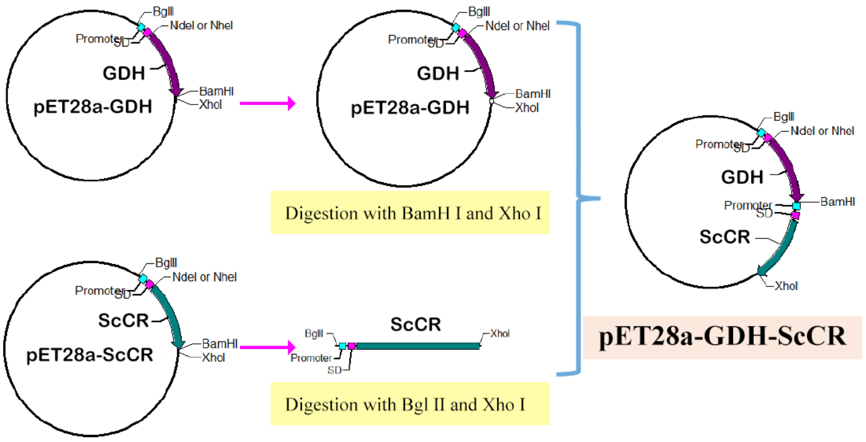

Scheme S1. Construction of recombinant pET28a_GDH_ScCR plasmid.

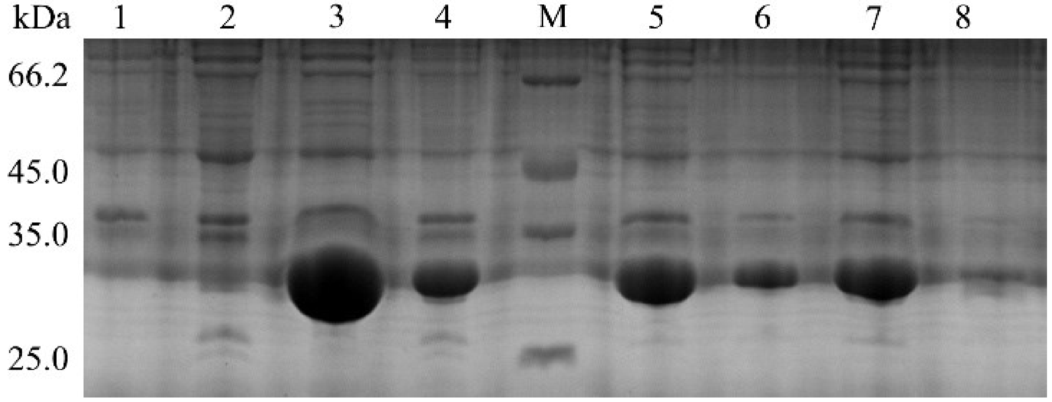

Fig. S3. SDS-PAGE analysis of protein present in the supernatant and precipitant
Lane M: protein marker; Lane 1: supernatant of *E.coli*/pET28a; Lane 2: precipitant of *E.coli*/pET28a; Lane 3: supernatant of *E.coli*_GDH_ScCR; Lane 4: precipitant of *E.coli*_GDH_ScCR; Lane 5: supernatant of *E.coli*_ScCR; Lane 6: precipitant of *E.coli*_ScCR; Lane 7: supernatant of *E.coli*_GDH; Lane 8: precipitant of *E.coli*_GDH

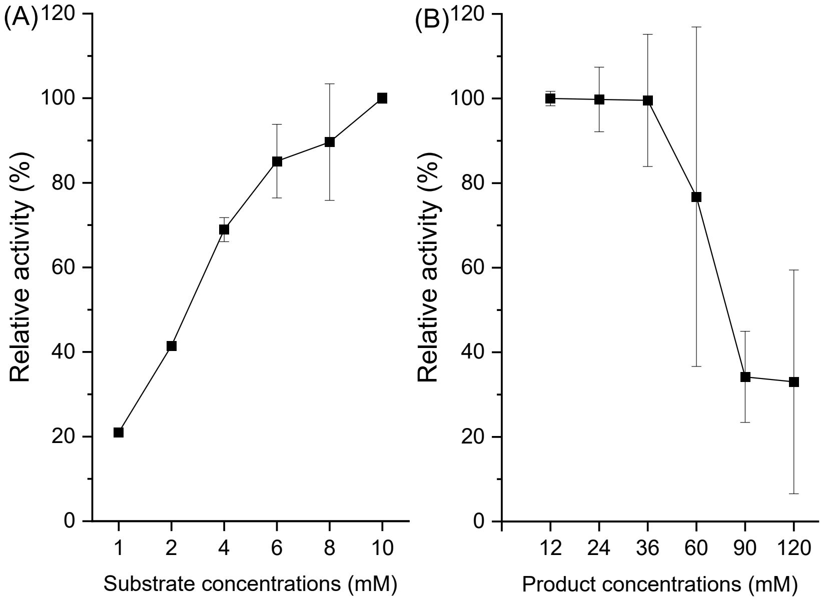

Fig. S4. Effect of substrate concentrations (A) and product concentrations (B) on ScCR-catalyzed synthesis of 3A5HEF. Conditions for Figure S4A: 1-10 mM 3A5AF, 1 mg/mL ScCR, 0.05 mg/mL GDH cell-free extract, 0.1 mM NADH, Mol$_{glucose}$: Mol$_{3A5AF}$= 2:1, 1 mL sodium phosphate buffer (0.1 M, pH 7.0) containing 10% DMSO (v/v), 35 °C, 150 r/min, 0.5 h; conditions for Figure S4B: 10 mM 3A5AF, 1 mg/mL ScCR, 0.05 mg/mL GDH, 0.1 mM NADH, 20 mM glucose, 1 mL sodium phosphate buffer (0.1 M, pH 7.0) containing 10% DMSO (v/v), 35 °C, 150 r/min, 12-120 mM 3A5HEF. The relative activities were based on the changes in the substrate concentrations after the reaction of 0.5 h.

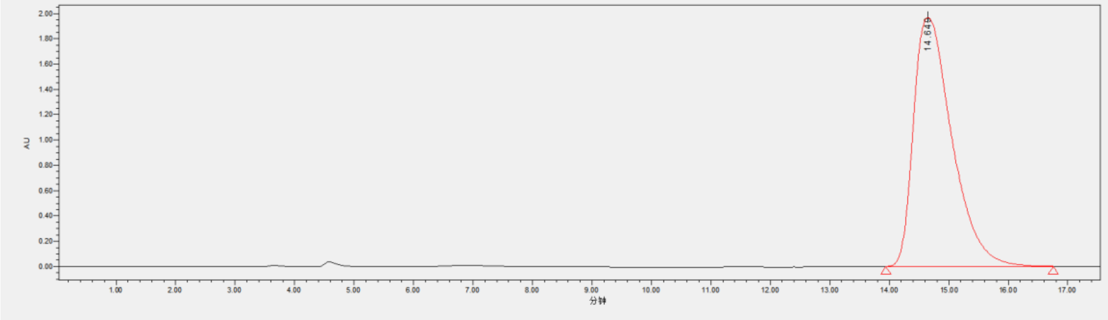

Fig. S5. HPLC analysis of (*R*)-3A5HEF obtained on a preparative-scale experiment. General conditions: the mobile phase: a mixture of acetonitrile and 0.4% $(NH_4)_2SO_4$ aqueous solution (pH 3.5, 5/95, v/v); flow rate: 0.6 mL/min.

1.1.1 Expression and purification of RalADH

The recombinant plasmid pET22b-RalADH was synthesized by Nanjing GenScript Biotechnology Co., Ltd. with codon optimization for *E. coli*. The enzyme expression was performed according to the method described previously (Kulig, et al., 2012, Lavandera, et al., 2008). Similarly, the enzyme purification steps were the same as those for purifying ScCR, with the exception of the elution buffer (300 mM imidazole, 500 mM NaCl, pH 7.0, 100 mM sodium phosphate buffer). The purified enzyme was subjected to SDS-PAGE analysis (Fig. S6).

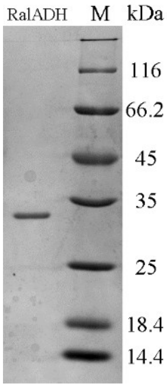

Fig. S6. SDS-PAGE analysis of purified RalADH. Lane M: protein marker

1.1.2 Expression and purification of AceCR

The expression of AceCR was performed according to the method previously described (Wei, et al., 2017). The enzyme purification steps were the same as the previous report (Wei, et al., 2017), with the exception of using pH 7.0 sodium phosphate buffer (instead of pH 6.5) in binding, elution and desalting buffers. The purified enzyme was subjected to SDS-PAGE analysis (Fig. S7).

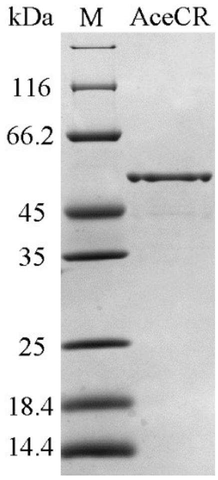

Fig. S7. SDS-PAGE analysis of purified AceCR. Lane M: protein marker

1.1.3 Expression and purification of SynADH

The expression of SynADH was performed as described by Jia et al (Jia, et al., 2019) with slight modifications in the induction conditions (0.5 mM IPTG at 20 ºC and 160 r/min). Besides, the purification steps of SynADH were the same as those for purifying ScCR. The purified enzyme was subjected to SDS-PAGE analysis (Fig. S8).

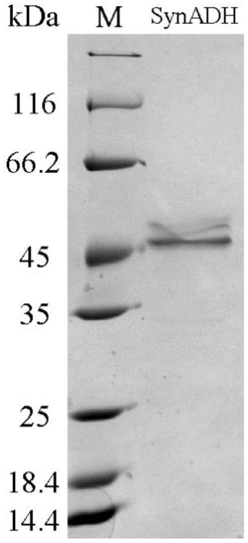

Fig. S8. SDS-PAGE analysis of purified SynADH. Lane M: protein marker

1.1.4 Expression and purification of HLADH

The expression of HLADH was performed as described by Jia et al (Jia, et al., 2019). Similarly, the enzyme purification steps were the same as those for purifying ScCR. The purified enzyme was subjected to SDS-PAGE analysis (Fig. S9).

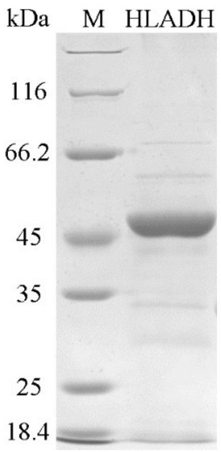

Fig. S9. SDS-PAGE analysis of purified HLADH. Lane M: protein marker

1.1.5 Expression and purification of ADH434 and AAD1669

Pichia pastoris X-33/pPICZα A-ADH434 and *P. pastoris X-33*/pPICZα A-AAD1669 (from *Meyerozyma guilliermondii* SC1103) were previously constructed by our laboratory (Xia, et al., 2020). Prior to use, cells were inoculated on YPD plates and cultivated at 30 °C for 3 d. A single colony was picked out and pre-cultivated in 30 mL BMGY medium at 30 °C and 230 r/min until the OD_{600} of the culture reached 2.0 ~ 6.0 (approximately 24-36 h). Upon centrifugation, the yeast cells were resuspended with 100 mL of BMMY medium, so that the OD_{600} of the culture was 1. The culture was cultivated at 30 °C and 230 r/min to induce the enzymes expression for approximately 3 d. Methanol was added every 24 h until its final concentration reached 1% (v/v).

Upon centrifugation (15285 × g, 20 min) at 4 °C, the supernatant was collected. At °C, ammonium sulfate powder was slowly added to 80% saturation, continue stirring for another 2 hours. After centrifugation for 15 min (15285 × g, 4 °C), the protein precipitates were collected. The precipitates were resuspended with pH 7.0 sodium phosphate buffer (100 mM). The crude enzyme solutions were obtained after dialysis and concentration. Fig. S10 shows SDS-PAGE analysis of the crude enzyme.

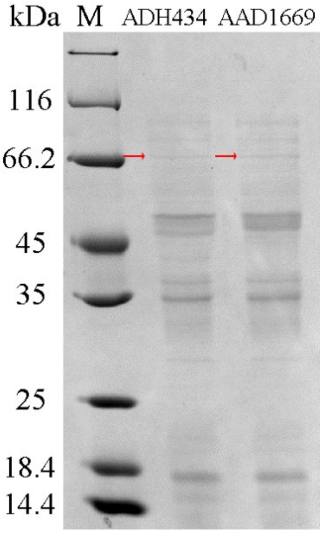

Fig. S10. SDS-PAGE analysis of crude ADH434 and AAD1669. Lane M: protein marker

1.2 Enzyme assay

Carbonyl reductase/alcohol dehydrogenase activities were determined by monitoring the changes in the absorbances at 340 nm within 3 min using a Shimadzu UV2550 spectrophotometer (Japan). The enzyme-catalyzed reduction of benzaldehyde/COBE was conducted with NAD(P)H at 30 °C in 0.4 mL sodium phosphate buffer (0.1 M, pH 7.0). One U corresponds to the amount of enzyme which oxidizes 1 μmol NAD(P)H per minute at 30 °C and pH 7.0. The specific activities of ScCR, YueD, RalADH, AceCR, SynADH and HLADH were presented in Table S2.

Table S2. Specific activities of various CRs/ADHs.

Entry	Enzyme	Substrate	Cofactor	Specific activity (U/mg)
1	ScCR	COBE	0.1 mM NADH	23.4
2	YueD	COBE	0.1 mM NADPH	1.1
3	RalADH	benzaldehyde	0.5 mM NADPH	0.2
4	AceCR	COBE	0.5 mM NADH	17.5
5	SynADH	benzaldehyde	0.5 mM NADPH	2.4
6	HLADH	benzaldehyde	0.5 mM NADH	6.6

Reaction conditions: 5 mM substrate, 0.1/0.5 mM NAD(P)H, 0.4 mL sodium phosphate buffer (0.1 M, pH 7.0), 30 °C

Table S3. Kinetic parameters of two enzymes using 3A5AF as a substrate.

Enzyme	k_{cat} (s^{-1})	K_m (mM)	K_{cat}/K_m (M^{-1} s^{-1})
ScCR[a]	0.1	6.3	15.9
YueD[b]	0.7×10^{-3}	0.5	1.4

[a]Reations were performed in sodium phosphate buffer (0.1 M, pH 7.0) at 25 °C; the native molecular mass of ScCR: 326 kDa. [b]Reations were performed in sodium phosphate buffer (0.1 M, pH 8.0) at 25 °C; the native molecular mass of YueD: 59.3 kDa.

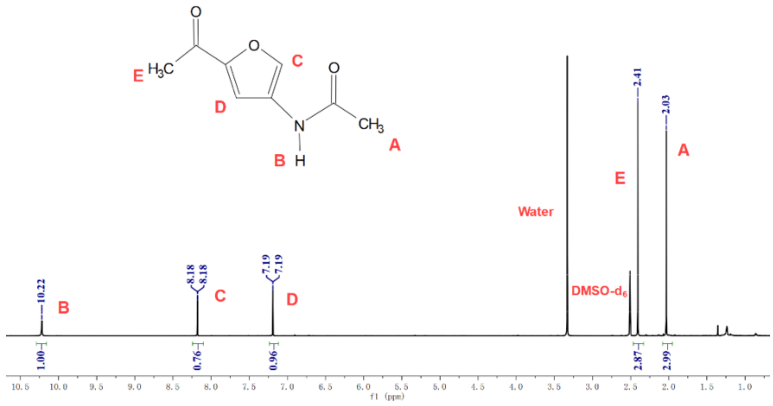

Fig. S11. ^{1}H NMR of 3A5AF (DMSO-d_6, 600 MHz)

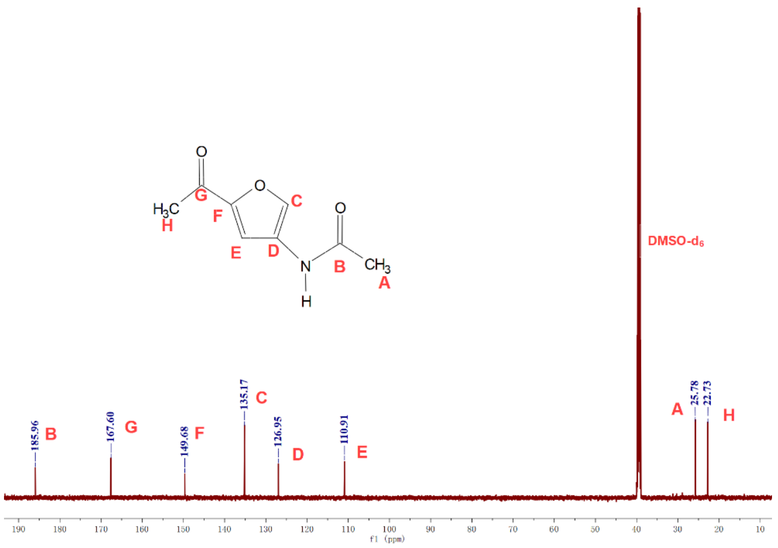

Fig. S12. ^{13}C NMR of 3A5AF (DMSO-d_6, 125 MHz)

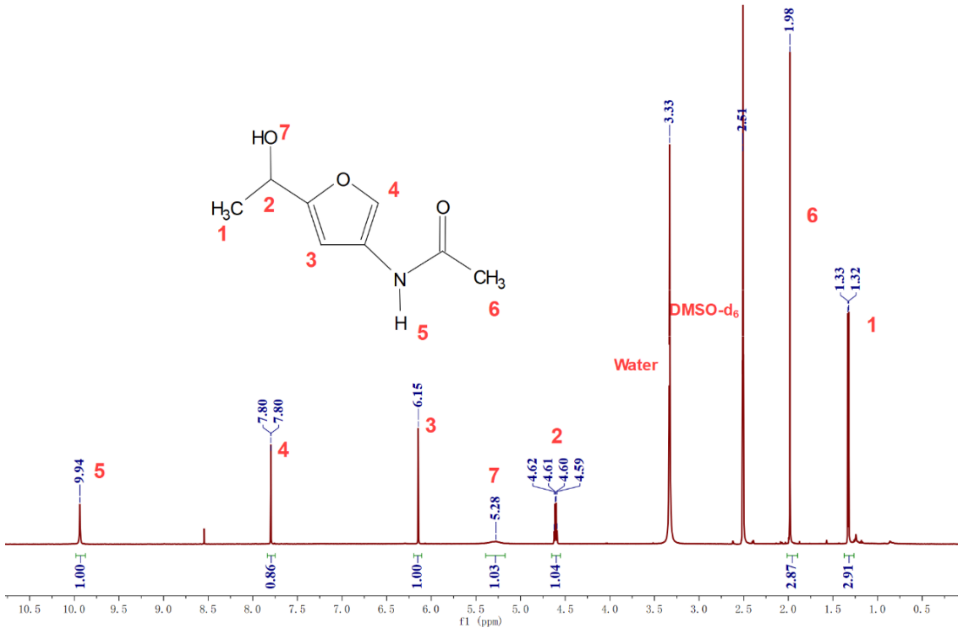

Fig. S13. ^{1}H NMR of 3A5HEF (DMSO-d_6, 600 MHz)

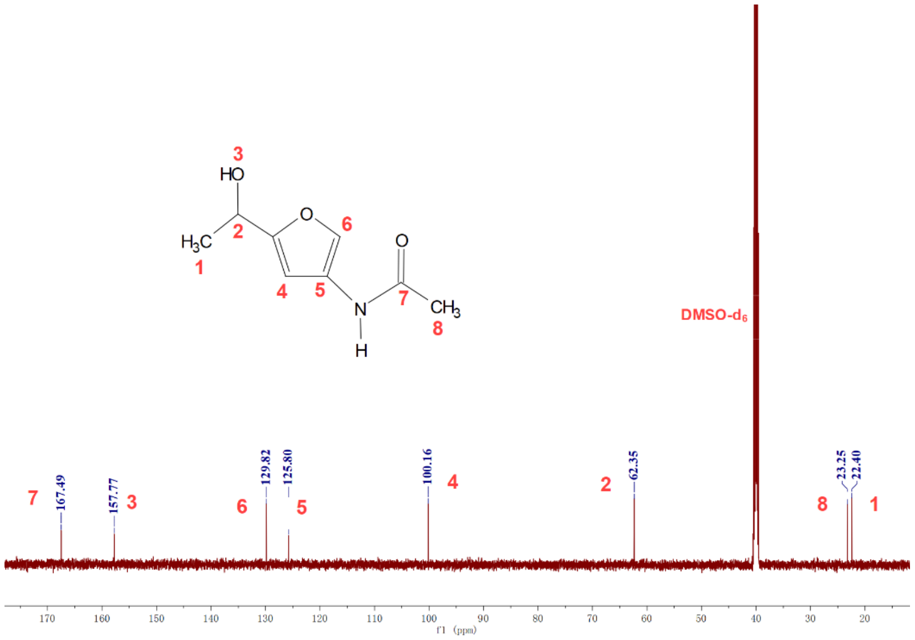

Fig. S14. ^{13}C NMR of 3A5HEF (DMSO-d_6, 125 MHz)

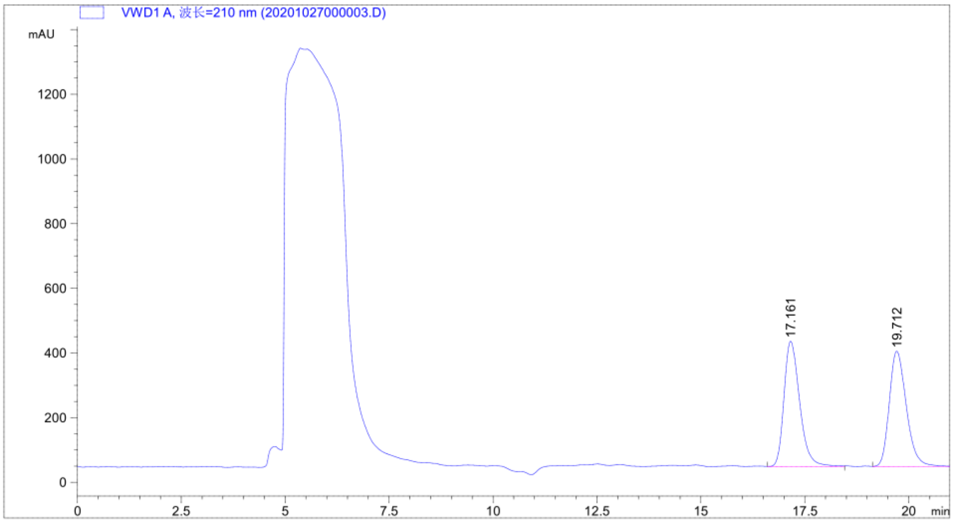

Fig. S15. The chiral HPLC spectrum of 3A5HEF prepared by chemical method. The retention time of the products as follows: *t* [(*R*)-3A5HEF] = 19.7 min, *t* [(*S*)-3A5HEF] = 17.2 min.

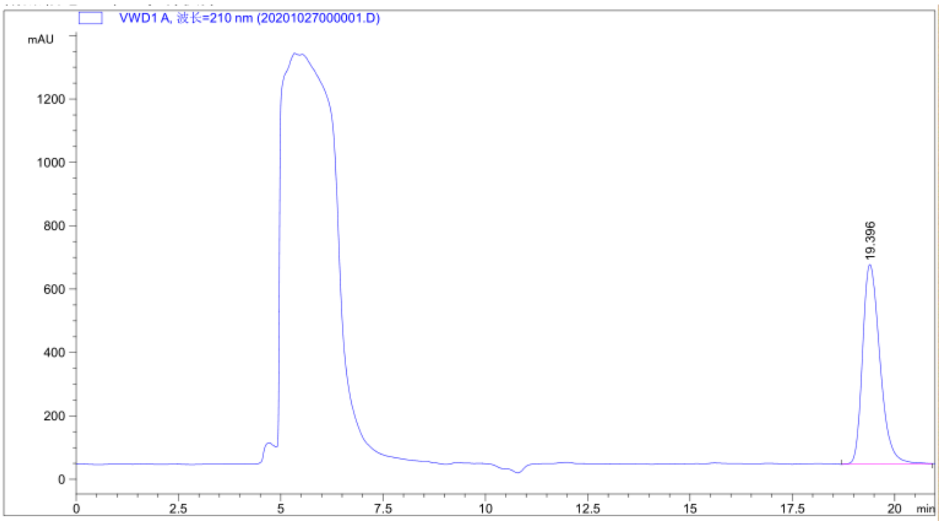

Fig. S16. The chiral HPLC spectrum of (*R*)-3A5HEF obtained on a preparative-scale experiment.

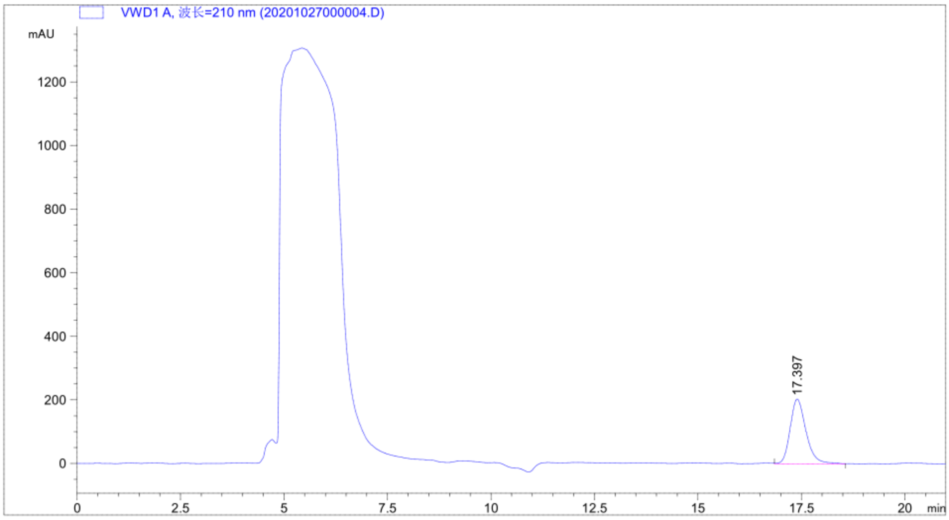

Fig. S17. The chiral HPLC spectrum of (*S*)-3A5HEF

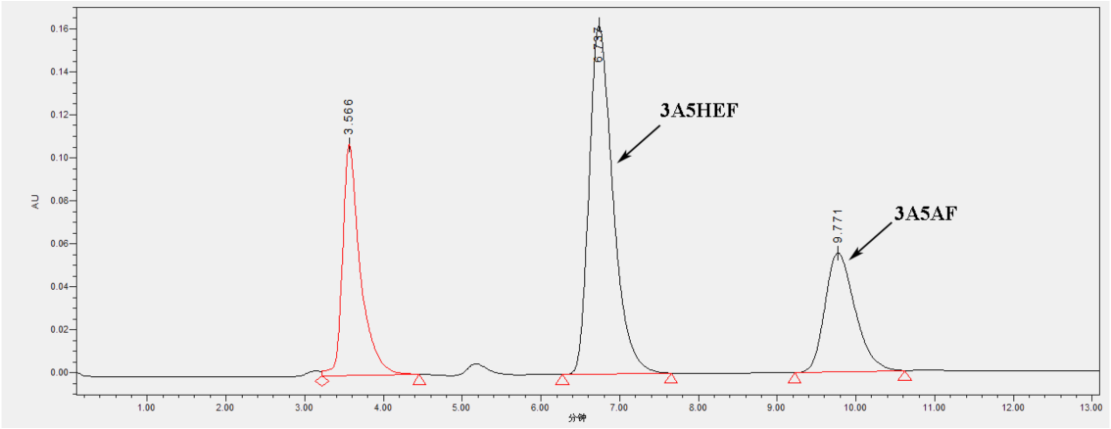

Fig. S18. HPLC analysis of the reaction mixture in enzymatic reduction of 3A5AF

General conditions: the mobile phase: a mixture of acetonitrile and 0.4% $(NH_4)_2SO_4$ aqueous solution (pH 3.5, 2/8, v/v); flow rate: 0.6 mL/min.

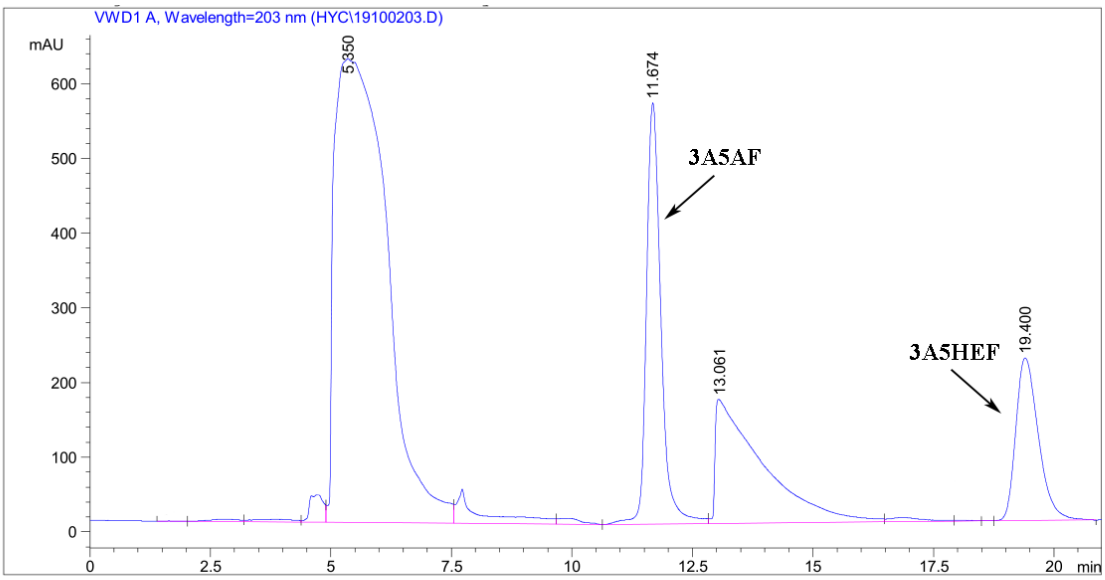

Fig. S19. The chiral HPLC spectrum of the reaction mixture in enzymatic reduction of 3A5AF to (*R*)-3A5HEF

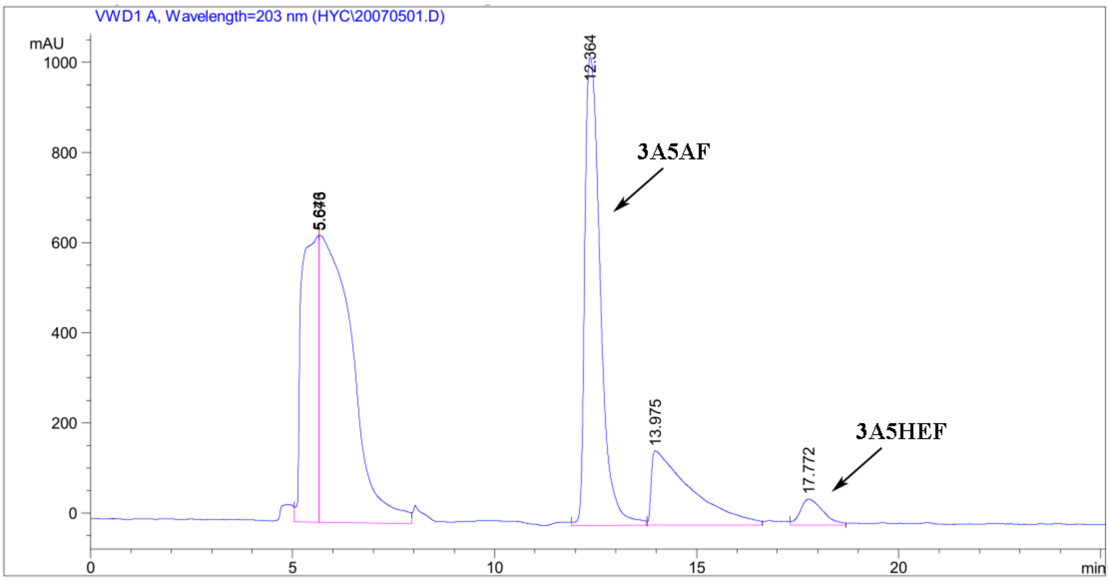

Fig. S20. The chiral HPLC spectrum of the reaction mixture in enzymatic reduction of 3A5AF to (*S*)-3A5HEF

RalADH gene sequence (Accession number: EU485985)

atgtatcgactattaaacaaaacagccgtcataaccggtggaaacagcggcattggcctcgccacagcgaagcgcttcgttgccgaggg
tgcctatgtattcattgtcggtcgccggcggaaggaactcgagcaggcggccgcagaaatcggtcggaatgtcacggcggtcaaagccga
tgtgacaaagcttgaagacctggaccgactttacgcgattgtgcgtgagcaacggggtagcatcgacgtactatttgcgaattccggcgcaat
cgagcaaaagacgcttgaggagattactccggaacactatgacaggactttcgatgtcaacgttcggggattgatcttcaccgtgcagaagg
cacttcctctgctgcgagacggcggcagcgtgatcctgacaagctcggtagccggcgtcctaggattacaggcgcacgacacgtatagtgc
cgccaaggcagcggtaaggtcgctcgcgaggacatggaccactgagttgaaaggtcgcagcattcgtgtcaacgcggtaagcccagggg

cgatcgacacgcctatcatagaaaaccaggtctctacacaggaagaagctgacgagctgcgtgcgaaatttgcagctgcgacgcccctgg gtcgcgtcggacgacctgaagagctggcagcggccgtgttatttcttgcatcggacgacagtagctacgtagccggcattgagctgtttgtg gacggtggattgacccaggtctaa

Uncategorized References

Kulig J, Simon RC, Rose CA, Husain SM, Häckh M, Lüdeke S, Zeitler K, Kroutil W, Pohl M, Rother D (2012) Stereoselective synthesis of bulky 1,2-diols with alcohol dehydrogenases. Catal. Sci. Tech. 2(8): 1580-1589. 10.1039/C2CY20120H

Lavandera I, Kern A, Ferreira-Silva B, Glieder A, de Wildeman S, Kroutil W (2008) Stereoselective Bioreduction of Bulky-Bulky Ketones by a Novel ADH from Ralstonia sp. J. Org. Chem. 73(15): 6003-6005. 10.1021/jo800849d

Wei P, Cui Y-H, Zong M-H, Xu P, Zhou J, Lou W-Y (2017) Enzymatic characterization of a recombinant carbonyl reductase from Acetobacter sp. CCTCC M209061. Bioresour. Bioprocess. 4(1): 39. 10.1186/s40643-017-0169-1

Jia H-Y, Zong M-H, Zheng G-W, Li N (2019) One-pot enzyme cascade for controlled synthesis of furan carboxylic acids from 5-hydroxymethylfurfural by H_2O_2 internal recycling. ChemSusChem 12(21): 4764-4768. 10.1002/cssc.201902199

Xia Z-H, Zong M-H, Li N (2020) Catalytic synthesis of 2,5-bis(hydroxymethyl)furan from 5-hydroxymethylfurfual by recombinant *Saccharomyces cerevisiae*. Enzyme Microb. Technol. 134: 109491. 10.1016/j.enzmictec.2019.109491
